# In the Thick of It: Formation of the Tuberculous Granuloma and Its Effects on Host and Therapeutic Responses

**DOI:** 10.3389/fimmu.2022.820134

**Published:** 2022-03-07

**Authors:** Mark R. Cronan

**Affiliations:** In Vivo Cell Biology of Infection Group, Max Planck Institute for Infection Biology, Berlin, Germany

**Keywords:** tuberculosis, granuloma, granuloma organization, host-directed therapies, macrophage reprogramming, macrophage

## Abstract

The defining pathology of tuberculosis is the granuloma, an organized structure derived from host immune cells that surrounds infecting *Mycobacterium tuberculosis*. As the location of much of the bacteria in the infected host, the granuloma is a central point of interaction between the host and the infecting bacterium. This review describes the signals and cellular reprogramming that drive granuloma formation. Further, as a central point of host-bacterial interactions, the granuloma shapes disease outcome by altering host immune responses and bacterial susceptibility to antibiotic treatment, as discussed herein. This new understanding of granuloma biology and the signaling behind it highlights the potential for host-directed therapies targeting the granuloma to enhance antibiotic access and tuberculosis-specific immune responses.

## Introduction

The granuloma has been recognized by pathologists as the defining pathology of tuberculosis for more than 100 years (1–3). This structure is organized around infecting *Mycobacterium tuberculosis*, the etiologic agent of tuberculosis, from host immune cells (2, 4). As the location of much of the bacteria within infected individuals, the granuloma pivotally shapes the way that *Mtb* interacts with the host immune system (2). This structure also helps define our clinical approaches to *Mtb* treatment, serving as a substantial barrier to *Mtb* chemotherapeutics (5, 6). In this review, I will discuss the organization of the tuberculous granuloma and the host and bacterial signaling that shape its formation. I will also consider the ways in which this central structure of tuberculosis contributes to the pathogenicity and clinical outcomes of this disease. As a pivotal structure to mycobacterial pathogenesis and treatment, the potential of targeting this structure as an adjunctive therapy and potential pitfalls of this approach will be considered.

## Early *Mtb* Infection and Granuloma Organization

*Mtb* is an obligate human pathogen, passing from host to host in the aerosols generated by the cough of an *Mtb*-infected individual (7). Droplets containing Mtb bacilli are deposited into the lungs of newly infected individuals where the bacteria are rapidly phagocytosed by resident alveolar macrophages (7). These macrophages then bring the bacterium out of the airway, carrying the bacterium into the interstitium of the lung. Once out of the airway, the bacterium is able to use the concerted action of many virulence factors to evade immune-mediated killing and efficiently replicate within populations of macrophages and other recruited immune cells (7–10). As the infection progresses, continued recruitment and aggregation of macrophages and other immune populations to the site of *Mtb* infection leads to the formation of the granuloma, the defining pathology of tuberculosis infection (2, 4, 11).

**Figure 1:**
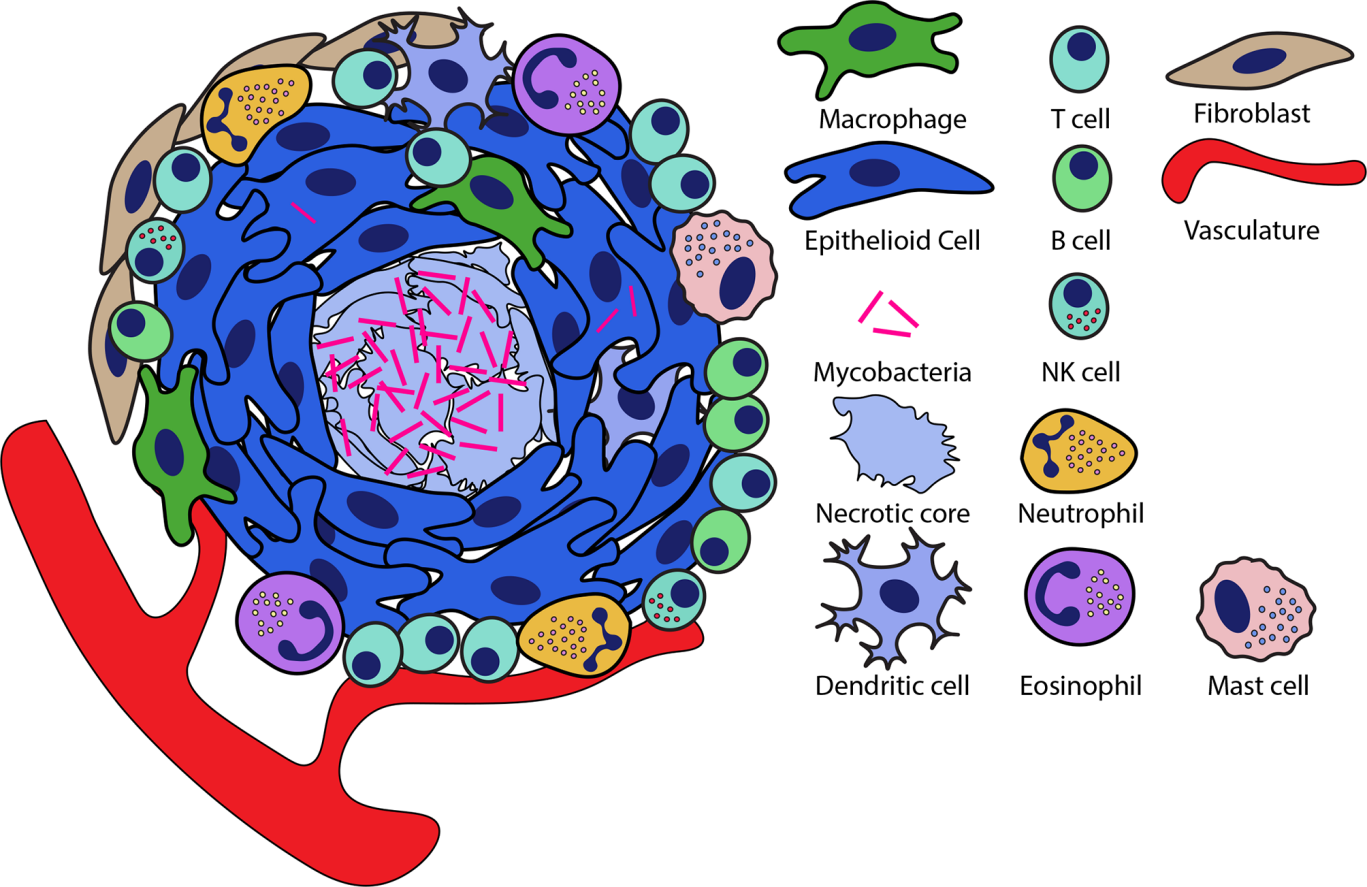
Organization of necrotic granulomas during mycobacterial infection. Necrotic granulomas are structured around a central core of necrotic cell debris in which much of the bacteria are concentrated. Layers of epithelioid macrophages surround the necrotic core interspersed with other macrophage populations. A diversity of other cell types are recruited to the granuloma and can be integrated into this structure at the periphery as well as within the epithelioid layers of the granuloma.

The macrophage is central to the formation of the granuloma. Macrophages comprise much of the cell population of the granuloma and form the inner layers of the granuloma, serving as a central scaffold around which the other cell populations are nucleated (2, 4, 11). While macrophage populations are highly motile under normal physiological conditions or infection, during granuloma formation, these cells undergo a pronounced morphological differentiation termed ‘epithelioid differentiation’ where they tightly interdigitate and aggregate with their neighbors to form the granuloma (4, 12). These epithelioid macrophages are a central characteristic of the tuberculous granuloma, but they are joined by other macrophage populations, including conventional macrophages, lipid-laden foamy macrophages and multinucleate macrophage giant cells (2, 13). Beyond macrophage populations, the granuloma is characterized by a broader immune response that recruits many other cell types to the granuloma. Cell populations recruited to the granuloma include myeloid populations such as neutrophils, dendritic cells, eosinophils, and mast cells, lymphocyte populations including T cells, B cells, NK cells and ILCs and nonhematopoietic cells such as fibroblasts, endothelial and epithelial cells (**Figure 1**) (2, 14–20).

Granulomas in *Mtb*-infected patients take many shapes and forms. Tuberculosis is most classically associated with the formation of caseous necrotic granulomas, wherein the central region of the granuloma has undergone necrotic cell death, leading to the formation of a core of cell debris that frequently have a soft, cheese-like consistency termed caseum (4, 5, 21). These necrotic granulomas come in many forms including fibrotic granulomas (encapsulated by a fibrotic rim), calcified granulomas (necrotic core is mineralized), and suppurative granulomas (necrotic core infiltrated by neutrophils) (5, 18). *Mtb* infection also results in the formation of cellular or non-necrotic granulomas, where the central necrotic core is absent and the bacteria reside intracellularly (5, 18). Furthermore, the primary granulomas that arise from initial infection events are also morphologically distinct from the post-primary granulomas that form *via* encapsulation of caseus pneumonia from bronchogenic spread of *Mtb* in individuals previously exposed to the bacterium (22). This text will largely focus on the primary granulomas that have been the recent focus of the field, however, with the newfound re-recognition of post-primary tuberculosis and animal modeling of this process (23), future studies comparing these lesions will be of great interest.

Given the diversity of granuloma types and the on-demand nature of granuloma assembly, there is considerable heterogeneity in the organization of individual granulomas (5, 24, 25). However, a general pattern of spatial organization is observed in the granuloma, wherein the central regions of the granuloma are macrophage-rich with many of these macrophages having undergone epithelioid transition, whereas lymphocyte populations tend to be largely confined to the periphery where they comprise a lymphocyte-rich cuff that surrounds the granuloma (**Figure 1**) (2, 18, 26). At the edges of the granuloma, these lymphocytes can be organized along with antigen presenting cells into tertiary lymphoid follicles (also called iBALT in lungs) that allow for local antigen presentation at the site of infection (27, 28). In necrotic granulomas, bacteria are found largely in the necrotic core of granulomas, while smaller numbers of bacteria live intracellularly largely within the macrophage-rich regions surrounding the core (18, 26). Similarly, in cellular granulomas *Mtb* resides predominantly in the central regions of the granuloma, though the absence of necrosis in these granulomas means that *Mtb* resides intracellularly, predominantly within the macrophage populations that comprise the core of these granulomas (5, 18).

Granulomas are commonly presented as a number of distinct granuloma types, but it is important to recognize that all of these distinct granuloma types can be observed within a single infected host (21, 24, 29). Individual bacteria within the host can experience radically different granuloma environments and local inflammatory states depending on the type of granuloma they are in, the age of the granuloma and the regional immune response (24). Throughout the course of infection in a single infected individual, these local differences mean that while some granulomas progress, expand and consolidate with neighbors, other granulomas that are established subsequently regress (24). This is supported by both classical and recent findings that have demonstrated that sterile, frequently calcified, lesions freely coexist in *Mtb* infected individuals along with active lesions containing large numbers of *Mtb* (24). This considerable heterogeneity between individual lesions not only underlies differences in the infection outcomes within each lesion, but also likely complicates the clinical treatment of tuberculosis, as individual lesions respond heterogeneously to antibiotic treatment.

Not only is there substantial variation between granulomas, but the organization of individual granulomas is considerably more complex than is generally depicted. Granulomas are commonly represented as circular structures (including in the figures herein). While this circular depiction of granulomas is true to what is commonly observed in histological sections and is also representative of many smaller lesions, classical work as well as recent PET-CT and micro-CT analysis of granulomas in human patients has found that larger granulomas form substantially more complex structures (30–32). These larger granulomas are highly elaborated and branched, factors that make granuloma organization difficult to assess by histology (30–32). While researchers have worked to characterize the local environment of granulomas, these extended structures mean that a single granuloma may instead sample a much more diverse variety of environments within the lung than widely expected (30). In particular, many of the larger, elaborated granulomas, while lodged in lung tissue, were found to directly access the airways in these patients, potentially enabling the bronchogenic spread of *Mtb* from these granulomas through the airways (30). This complex, branched structure of the granuloma further suggests that even within a single granuloma, there may be substantial variation in local environment depending on where the bacteria are located within the granuloma, which may result in spatially distinct selective pressures even within a single granuloma.

While the spectrum of granuloma types has long been a hallmark of the human disease, granulomas are difficult to study in preclinical animal models due to discrepancies in granuloma morphology between models and human patients and species-specific limits in tool availability. The most widely used *Mtb* model are inbred mouse models such as C57BL/6 and BALB/c mice, which possess an expansive suite of immunological tools (33). However, these inbred mouse models fail to form the necrotic granulomas that define human disease (33, 34). Limitations of inbred mouse models have led to the development of new mouse models including C3HeB/FeJ mice (35), the C57BL/6 NOS2^-/-^ ear infection model (36), CBA/J IL10^-/-^ mice (37) and genetically diverse panels of mice, which develop necrotic lesions that more closely resemble the human disease (38, 39). However, the complex genetic backgrounds of these animals complicate the use of existing mouse genetic tools. Beyond mice, a number of other small mammal models have long been used as effective tuberculosis surrogate models, most notably rabbits and guinea pigs (33). These models phenocopy many features of human *Mtb* infection and granuloma formation, but also are restricted by tool availability and the limited use of genetic approaches in these models. The development of non-human primate models has allowed the interrogation of tuberculosis pathogenesis in a model that closely resemble human disease (29, 40), but cost, time, and ethical concerns limit the widespread adoption of this model. Outside of mammalian systems the zebrafish has emerged as a genetically tractable model to study mycobacterial granuloma formation (41–43). In this model, zebrafish are infected with *Mycobacterium marinum*, a close relative of *Mtb* that conserves many virulence factors with *Mtb* and is a natural pathogen of zebrafish (41). Infection of zebrafish can be performed within optically transparent zebrafish larvae to enable visualization of immune-mycobacterial interactions and early granuloma formation in live animals. Adult zebrafish can also be infected, resulting in a long-term infection and formation of necrotic granulomas that closely resemble the granulomas seen in human disease (42, 43). Findings in zebrafish have been predictive of human *Mtb* infection outcomes (44–48). However, as a surrogate model, there are several caveats to the zebrafish/*M. marinum* system including the use of *M. marinum* rather than *Mtb* itself, the degree of conservation of immune genes between humans and zebrafish and the absence of lungs in zebrafish. While many animal models exist for *Mtb* infection, each of these models have distinct strengths that allow researchers to match their question to an optimal model.

## Molecular Reprogramming of Macrophages and Host Determinants of Granuloma Formation

A central feature of mycobacterial granuloma formation in all of these models of tuberculosis is the epithelioid transformation of their macrophage populations. The term ‘Epithelioid cell’ was chosen by early pathologists due to the close resemblance of these macrophage populations to epithelial tissues (2, 4). Morphologically, these cells are characterized by a spread morphology, an elongated nucleus and tight interdigitation with surrounding macrophages (4, 11). The strikingly altered morphology and behavior of epithelioid cells compared to parental macrophages suggests that these cells undergo substantial reprogramming events during epithelioid transformation, but the nature of these reprogramming events remained unknown for over a century.

**Figure 2:**
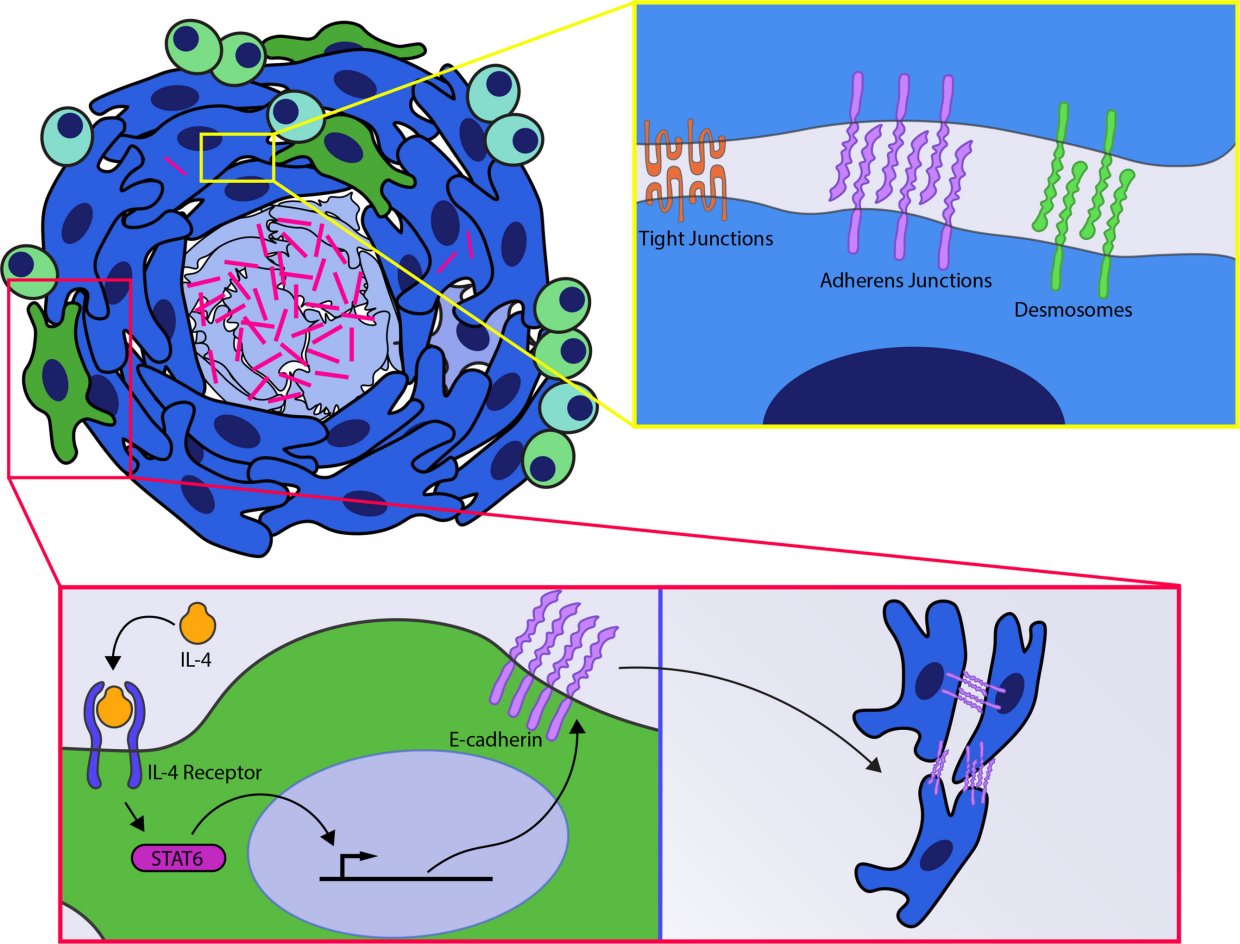
Host signals involved in granuloma formation. (Yellow box) Signaling events within epithelioid macrophages results in induction of epithelial cell-cell adhesion pathways including induction of adherens junctions, tight junctions and desmosomes within the macrophages of the granuloma. (Red box) IL4R signaling *via stat6* is required for induction of E-cadherin within macrophages and necrotic granuloma formation.

Using the zebrafish model, it was found that these granuloma macrophages undergo a bona fide epithelialization event during granuloma formation, broadly engaging epithelial-specific genes in a macrophage-epithelial transition that is analogous to mesenchymal-epithelial transition (46). Among the many characteristic rearrangements of epithelial tissues, these epithelioid cells formed functional E-cadherin-positive adherens junctions, desmosomes and tight junctions (**Figure 2**), alter their cytoskeletal structure and induce apical-basal polarity pathways (46). Engagement of this epithelialization program was essential to granuloma formation, as disruption of epithelialization by macrophage-specific expression of a dominant negative E-cadherin blocks granuloma formation and leads to the loss of epithelioid morphology (46). These rearrangements were observed not only in zebrafish, but also in samples from human patients by histological staining approaches (46, 47). Extensive engagement of epithelial markers could also be observed in mass spectrometry data sets from human granulomas, which further supports that epithelialization is an evolutionarily conserved feature of mycobacterial granulomas (49, 50). Beyond tuberculosis, E-cadherin induction has also been seen in schistosome granulomas as well as in dermal granulomas formed in sarcoidosis and foreign body reactions (51–55). The identification of epithelialization hallmarks within these diverse granuloma types also supports a wider involvement of epithelialization within granulomatous diseases generally.

That the macrophages of the granuloma undergo such profound cellular and molecular changes during granuloma formation raises the question of what factors could be driving these changes within macrophage populations. As central mediators of the immune response, cytokine signals are likely candidates for regulating granuloma formation. Classically, *Mtb* granulomas have been thought to be driven by type 1 immune responses (50, 56). Amongst type 1 cytokines, IFN-γ and TNF have been most closely associated with *Mtb* pathogenesis and granuloma formation. Both IFN-γ and TNF are crucial to the control of *Mtb* burden during infection in preclinical animal models and human patients (57–64). Loss of these cytokines has also been linked to altered granuloma architecture during *Mtb* infection, suggesting that signaling through these pathways may be required for ordered granuloma formation (61–64). However, in the case of TNF, subsequent longitudinal imaging experiments in zebrafish revealed that TNF deficient animals have normal granuloma formation early in infection, and that loss of granuloma morphology was instead due to the accelerated cell death caused by exuberant bacterial growth in these animals (65). Further evidence that TNF is required for bacterial restriction but not granuloma formation has been found in macaques, where neutralization of TNF with anti-TNF antibodies resulted in normal granuloma formation but markedly enhanced bacterial burden (66). Similar results have been observed for IFN-γ in mice infected with the attenuated vaccine strain M. bovis BCG, where IFN-γ-deficient animals infected with BCG still form similar numbers of morphologically normal granulomas compared to wildtype controls (67). Additionally, while C57BL/6 mice fail to form necrotic granulomas, disseminated infection in C57BL/6 mice deficient for the critical IFN-γ-induced factor iNOS resulted in the formation of sporadic necrotic granulomas that could be enhanced by neutralization of either IFN-γ or TNF (36). Therefore, while type 1 pathways are important for *Mtb* restriction, it is less clear that they are required for granuloma architecture, suggesting that other cytokine signaling pathways may be required for granuloma formation.

While *Mtb* pathology has commonly been thought to be driven by type 1 immunity, in Schistosome granulomas it has long been known that type 2 immunity is crucial to driving formation of the granuloma structure (12, 68). Type 1 and type 2 immunity are thought to drive substantially divergent transcriptional programs in macrophages (69). It seems unlikely that such disparate transcriptional programs could drive the formation of the same complex and highly organized granuloma structure. Instead, it is possible that the *Mtb* granuloma is driven by type 2 immunity and the degree of type 2 polarization within the tuberculous granuloma has been underestimated.

A growing number of studies have observed that there is a substantial type 2 inflammatory response during *Mtb* infection, seen in the lung, and within the granulomas in *Mtb* patients, as well as animal models that form necrotic granulomas (18, 47, 70–73). Owing to the identification of these type 2 responses in human disease, efforts have been made to model the involvement of these cytokines in infection and granulomatous responses. Due to the availability of genetic tools, these experiments were initially done in inbred mouse models deficient for type 2 signaling. However, in these models, loss of type 2 signaling had only mild effects on granuloma formation and modest to moderate effects on bacterial burden, suggesting that type 2 immunity was largely dispensable for granuloma formation during *Mtb* infection (74–76). One confounding point to these experiments is that the C57BL/6 and BALB/c mouse model used in these studies do not form the highly organized necrotic granulomas seen in human disease. Recognizing that the strong type 1 environment observed in many *Mtb* infected inbred mouse lines may limit the involvement of type 2 immune responses in these models, Heitmann et al. used transgenic mice overexpressing IL-13 to instead look at how enhanced type 2 responses within mice would alter the pathology and trajectory of *Mtb* infection (77). In contrast to non-transgenic C57BL/6 littermates, IL-13 overexpressing animals infected with *Mtb* formed organized necrotic granulomas that closely resembled human disease, suggesting that type 2 pathways contributed to necrotic granuloma formation (77). Necrotic granuloma formation was accompanied by increased bacterial burden in these animals, suggesting that either the enhanced type 2 signaling or granuloma formation itself was bacterial beneficial (77).

Further evidence for the role of type 2 immunity in mycobacterial granuloma formation has come from recent studies on macaques and zebrafish. Using histological staining of macaque granulomas and scRNA-seq analysis of the organized necrotic granulomas that form in zebrafish it was found that the epithelialized macrophage populations in the granuloma had increased type 2 inflammatory signaling (47). These type 2 responses were required for necrotic granuloma formation and epithelialization (**Figure 2**), as zebrafish deficient for type 2 signaling by deletion of either the IL-4 receptor or the downstream transcription factor *stat6* failed to undergo macrophage-epithelial transformation and no longer formed necrotic granulomas (47). Loss of type 2 signaling was associated with increased bacterial burden in *stat6*-deficient animals, raising the possibility that elevated bacterial burden could be driving necrotic granuloma breakdown (47). To test this hypothesis, mixed marrow chimeras possessing both wildtype and *stat6*-deficient cell populations were used to assess whether *stat6* functioned cell autonomously. Infection of these mixed marrow chimera animals resulted in the formation of necrotic granulomas. However, these necrotic granulomas were predominantly composed from the wildtype donor tissue (47). Despite the presence of organized necrotic granulomas in these animals, stat6-deficient populations failed to undergo epithelialization and were largely excluded from these granulomas, localizing instead to the periphery of these structures (47). Further supporting that type 2 signaling is required for granuloma organization independent of its burden effects, an ex vivo culture model (78) was used to test the effects of *stat6* inhibitors on established granulomas ex vivo. Addition of a *stat6* inhibitor to isolated granulomas reversed granuloma epithelialization and drove disaggregation of these structures, suggesting that continued *stat6* signaling was required to maintain granuloma organization (47). Thus type 2 responses appear to play a critical role in the organization of necrotic granulomatous structures. At later stages of infection, it is thought that erosion of these lesions into the airway can drive transmission in the process of cavitation (5, 21). Interestingly, work in human patients has indicated that elevated type 2 signaling is also associated with cavitation and transmission, suggesting that type 2 signaling may play crucial roles in multiple phases of the *Mtb* life cycle (71, 79).

While these studies have implicated type 2 immune responses driven by IL-4 and IL-13 in granuloma pathology, there are still a number of outstanding questions about how, when and where these cytokines are produced. In zebrafish, scRNA-seq demonstrated that IL-4 and IL-13 were produced by T cell and eosinophil populations within the granuloma (47). By contrast, in macaques, scRNA-seq found that IL-4 and IL-13 were particularly highly produced by recruited mast cells, indicating that there may be some species specific variation in the cell types that are producing type 2 cytokines (15). Beyond IL-4 and IL-13, it seems likely that other cytokines and signals are also required to drive necrotic granuloma formation, as elevated levels of IL-4 and IL-13 are observed in many inflammatory responses that do not lead to granuloma formation (80). The identity of these potential accessory factors is unknown, but experiments characterizing the induction of the epithelial cell-cell adhesion molecule E-cadherin in macrophages in culture found that E-cadherin induction by IL-4 and IL-13 could be enhanced by costimulation with IL-10 and TGFβ (52), suggesting that these two cytokines may act as accessory factors, facilitating granuloma formation through potentiation of the macrophage-epithelial transitions that underlie granuloma formation (46). Similarly, cell-cell interactions during infection and granuloma formation may also contribute to type 2 polarization and epithelioid transformation. Coculture of macrophages with platelets has found that platelets drive these macrophages into an M2 phenotype that is accompanied by the acquisition of epithelioid-like characteristics (81).

## Bacterial Determinants of Granuloma Formation

The granuloma has frequently been thought of as a host driven structure. However, studies of bacterial growth in mycobacterial infected hosts have found that bacterial expansion actually coincides with the formation of this structure (82, 83). The connection between bacterial growth and granuloma formation suggests that the mycobacteria themselves benefit from driving this structure (82, 83). The attenuated vaccine strain *M. bovis* BCG has previously been used to identify regions of the mycobacterial genome that are potentially involved in virulence (84–86). The ΔRD1 region of *M. bovis* was identified as a particularly crucial region that was lost in BCG strains and necessary for virulence (84, 85, 87). This region is broadly conserved in pathogenic mycobacterial species; mycobacterial species lacking the ΔRD1 region are strongly attenuated *in vitro* and *in vivo* (82, 87, 88). Studies of ΔRD1 *M. marinum* have found that this region is necessary for effective granuloma formation either by innate immunity alone in larval zebrafish or in the context of both innate and adaptive arms in adult zebrafish (42, 82, 83). These defects were associated in part with decreased recruitment of macrophages and led to the formation of smaller, less well-organized granulomas (83, 89). In larval zebrafish, the crucial role for the ΔRD1 region in mycobacteria was found to involve the secreted protein ESAT6, which is encoded within this region (89). ESAT6 was released from infected macrophages and was subsequenty taken up by the surrounding epithelium, where it drives expression of the chemotactic metalloprotease MMP9 within these epithelial populations (89). Elevated MMP9 levels were found in turn to lead to the recruitment of surrounding macrophages to the granuloma, facilitating granuloma growth and expansion (89).

Beyond secreted bacterial effectors, mycobacteria are known to produce a number of lipid species that are critically important to the ability of mycobacteria to influence host immune responses (90). This was demonstrated in classical experiments which found that injections of killed mycobacteria, particularly when emulsified within Freund’s adjuvant (so called Complete Freund’s Adjuvant) could organize a robust granulomatous response, suggesting that lipids or other heat-stable mycobacterial fractions were important mediators of the granulomatous response (91, 92). Early efforts to identify crucial lipids by Sabin identified three distinct lipid fractions, all which could induce varying levels of epithelioid and giant cell differentiation and granuloma formation within rabbits (91, 93). Subsequent work in mice identified the mycolic acids, particularly Trehalose 6, 6’-Dimycolate (TDM), as a major mediator of epithelioid transformation and granuloma formation (94–96). Specifically these studies found that TDM when emulsified in incomplete Freund’s Adjuvant or TDM coated beads was sufficient to induce epithelioid granuloma formation *in vivo* (94, 95, 97). Interestingly, experiments in mice found that the effects of TDM depend on the particle size of emulsions and the density of TDM on the surface of beads, which together with studies on TDM organization *in vitro*, suggested that TDM geometry on the surface of mycobacteria is crucial to the granulomatous response (95, 98, 99). Importantly, removal of cell surface TDM and other mycolic acids and lipid species by lipid extraction abrogates granuloma formation in mice, instead leading to a predominantly neutrophilic response with minimal macrophage involvement (94, 100). However, these findings with delipidated bacteria are complicated as these bacteria are rapidly removed by host immune responses (94, 100).

Taken together with the previous section, orderly formation of granulomas requires the concerted action of both host and bacterial responses. The granuloma has long been presumed to be a host-protective response, containing and walling off the mycobacteria within this structure. This effective, host beneficial response can be observed in the sterile calcified granulomas seen in human patients and animal models (24). However, given the long-term coevolution between *Mtb* and humans, the granuloma likely represents a stalemate, affording benefits for not only the host but also the pathogen. This view is further supported by the recognition that bacteria also drive this structure with conserved mediators to facilitate their expansion (82, 89, 94, 96). While aspects of the granuloma may benefit both host the bacterium, as we discuss in the following sections, this structure critically shapes the immune responses to mycobacteria and the clinical treatment of infection.

## Granuloma Formation Limits Immune Recognition of Infecting Mycobacteria

The hallmark of the granuloma is its complex three-dimensional organization of immune cells into the granulomatous structure. Despite the *ad hoc* assembly of granulomas by the host immune system, this process results in a generally reproducible spatial organization, with bacterial populations largely constrained to the central region of the structure, while distinct immune populations are distributed in the surrounding cellular layers (18). With the presence of so many bacteria in the core regions of this structure, effective immune responses must be mounted through the complex and polarized cellular and inflammatory milieu of this structure (18, 26, 49). Many immune populations require close contact with infecting mycobacteria to restrict these pathogens; the spatial distribution of bacteria and immune populations within the granuloma suggests that the highly structured granuloma could serve as a barrier to mycobacterial recognition, critically shaping the interaction of bacteria with the host immune system. Recent work described below, has supported this notion, finding the cellular environment of the granuloma plays a crucial role in shaping host immune responses.

While CD4+ T cells are clearly critical to control of *Mtb* infection, the persistent survival of *Mtb* in actively infected individuals demonstrates that these responses are ultimately unable to clear the infection in many individuals (101). One possible explanation for this disconnect is that the complex environment of the granuloma is able to shape and limit effective T cell responses. In mouse models, it was found that direct interaction between CD4 T cells and infected macrophages is critical to effective T cell help in Mtb infection (102). However, T cells are predominantly located at the periphery of the granuloma, distant from the bacteria in the myeloid-rich core regions of the granuloma (18, 26). Thus, the inability for T cell responses to completely eliminate *Mtb* in infected patients appears to be, at least in part, due to the spatial restriction of T cells to sites distant from infected macrophages (26). Similarly, both NK cells and CD8 T cells are known to be recruited to granulomas in humans and animal models of *Mtb* infection (15, 103, 104). CD8 cells, have been found to be crucial to control of *Mtb* burden in the mouse model *in vivo* (105, 106). NK cells by contrast have been demonstrated to be important for *Mtb* control in T cell-deficient animals, suggesting that these cells can also participate in functional responses to *Mtb* (107). Aspects of the responses of both of these cell types require close contact between the immune cells and *Mtb*-infected cells (108, 109), suggesting that granuloma architecture may also play a critical role in the *Mtb*-specific responses of these populations as well. In the case of T cells, the importance of spatial separation between these cells and bacterial populations is also supported by computational modeling of tuberculous granulomas, which indicated that the spatial organization of the granuloma was one of the major drivers limiting T cell responsiveness in the granuloma (110).

The factors driving the pronounced spatial separation between T cells and macrophages are likely complex and multifactorial, but some potential mechanisms contributing to this asymmetry have been identified. Intravital microscopy of *Mtb*-specific T cells in mouse granulomas demonstrated that these cells largely fail to arrest within the granuloma as *Mtb* antigens appear to be poorly recognized within the granuloma (111). The limited recognition of mycobacterial antigens within the granuloma is not due to failure by the macrophages to sense these antigens as administration of exogenous antigens led to rapid arrest of T cell populations (111). Lack of recognition of *Mtb* antigens within the granuloma may be due to repression of antigen presentation by *Mtb* within infected macrophages (112, 113) or by the failure of T cells to recognize specific *Mtb* antigens within infected macrophages (114). In mice and cell culture models, infected macrophages have also been found to export *Mtb* antigens to surrounding cells, which limits the ability of T cells to directly recognize infected cells within granuloma and which could also potentially lead to arrest of T cells at sites distant to infected cells (115).

Organized granuloma formation may also diminish immune cell recognition of *Mtb* by physical means. This could potentially result from macrophage reprogramming and induction of epithelial modules within the granuloma which may in turn limit the ability of immune populations to recognize or migrate to bacteria within the central regions of the granuloma. Supporting this idea in zebrafish, partially disrupted granulomas formed by mosaic expression of dominant negative E-cadherin, neutrophils are preferentially recruited to the areas of the granuloma deficient for epithelial transformation, suggesting that epithelialization limits recruitment of these cells (46). Similarly, the physical packing of the individual cells in the granuloma may also constitute a barrier to immune recognition in this context. In intravital imaging experiments of BCG liver granulomas in mice, it was found that T cell recruitment into the granuloma required morphological rearrangement of granuloma macrophages to accommodate incoming T cells (116). While the degree to which these physical constraints serve to limit mycobacterial recognition is still to be fully investigated, at least in the case of epithelialization-dependent neutrophil exclusion, altered immune access confers a bacterial beneficial role, as epithelialization-deficient animals had increased bacterial burden (46). This suggests that altering granuloma epithelialization or otherwise loosening the tight apposition of immune cell populations within the granuloma could potentially improve antimycobacterial immune responses. Illustrating the potential of therapies targeted at granuloma structure, work in macaques has found that IDO inhibition can reorganize granulomas, resulting in increased numbers of T cells at the core of the granuloma and decreased bacterial burden (117). Outside of cellular response, it could also be expected that loosening of the granuloma would facilitate the access of other immune mediators like antibodies. B cells are known to be recruited to granulomas in humans and animal models (28, 118, 119), although the effects of B cells and antibodies on infection have been varied despite the effective recognition of *Mtb* by B cell produced antibodies (120).

Beyond physical separation of immune cell and bacterial populations, the highly organized granuloma environment alters host immune responsiveness through the generation of local inflammatory environments that shape the cell signaling and responses of cells within the granuloma. Histological and mass spectrometry characterization of human and macaque granulomas found that there is a pronounced type 1 inflammatory signature in the central regions of the granuloma, presumably due to recognition of adjacent bacteria within the necrotic core while more distal regions expressed an anti-inflammatory signature (18, 47, 49). Immune markers were found to correlate with the local cytokine environment, as mass spectrometry studies indicated that the caseum is specifically enriched for the type 1 inflammatory cytokine TNF, eicosanoids and downstream components of IFN signaling (49). Studies in cell culture have indicated that TNF is induced by macrophages in response to mycobacteria and mycobacterial products (121, 122), suggesting that this local TNF signal could result from interaction of macrophages with mycobacterial products at the edge of the caseum. Local expression of type 2 immune mediators can also shape the granuloma environment. TGFβ, a potent type 2 cytokine, has long been known to alter immune responses by driving anti-inflammatory programs in diverse immune populations (123). Elevated levels of TGFβ are found in the plasma of individuals with active *Mtb* infection (124). Interrogation of the local inflammatory environment within granulomas in *Mtb* infected mice and macaques also found that there was a pronounced TGFβ signature within the granuloma (125). Similarly, using histological techniques and mass spectrometry, elevated TGFβ signaling and induction of downstream genes have recently been described in human patient samples as well (103). Using TGFβR-deficient mice, TGFβ was found to act in a T cell intrinsic fashion to repress Th1 differentiation within the granuloma (125). Loss of TGFβ signaling led to increased IFN-γ production by granuloma T cells and reduced bacterial burden in these animals (125).

The anti-inflammatory cytokine IL-10 has also been studied during *Mtb* infection, particularly within the granuloma. Computational studies have suggested that IL-10 may play a crucial role in maintaining a host-detrimental anti-inflammatory environment within the granulomas (126). Short-term studies in macaques indicated that while neutralization of IL-10 had no effect on bacterial burden, it altered the cytokine environment within the granuloma and led to increased fibrosis throughout the structure (127). While ongoing studies will continue to identify the specific cytokines that shape the granuloma, it is clear that the granuloma creates a constrained space in which cytokines can generate a local inflammatory environment to shape the host response to *Mtb*.

## The Granuloma Shapes Antibiotic Access and Efficacy for the Bacteria Within

That tuberculosis persists in the face of effective antibiotic therapies is a testament to the difficulty in clinically treating tuberculosis. As a slow growing bacterium, treatment necessitates the use of combination therapies composed of multiple antibiotics over extended time frames of 6 months or longer (128). In particular, the use of combination therapies is essential to prevent the acquisition of antibiotic resistance by *Mtb*. Despite these safeguards, antibiotic resistance is a growing problem, particularly due to difficulties in maintaining these complex drug regimens in resource-limited settings. This has led to the emergence of multidrug-resistant (MDR) and extensively drug-resistant (XDR) strains, which in turn have substantially longer and more complicated treatment regimens and greatly increased mortality (129). While many factors contribute to the complexities of treating tuberculosis in the clinic, the granuloma has been found to be a crucial structure that shapes antibiotic access to the bacterium and the responsiveness of the bacteria contained within.

Antimycobacterial antibiotics are first transported to their site of action *via* the host vasculature (6). *Mtb* granulomas, while largely composed from host immune populations, also recruit host vasculature to the nascent granuloma as observed in patient samples as well as animal models of infection (130, 131). The vascularization of granulomatous tissue is the result of angiogenic signals produced by the granuloma itself (19, 130, 132, 133). While multiple angiogenic factors are produced by the immune populations of the granuloma, the growth factor VEGF, predominantly produced by the macrophages of the granuloma, is crucial for efficient granuloma vascularization in both zebrafish and rabbits (130, 132, 134). In zebrafish, VEGF production by granuloma macrophages and angiogenesis is driven at least in part by recognition of bacterial TDM by these macrophage populations (134). However, there is considerable heterogeneity in the degree of vascularization between individual granulomas, which may restrict drug delivery to certain lesions (5, 30, 131). In addition, despite the strong vascularization of certain lesions, the improvised nature of the vasculature formed around the granuloma may limit the degree to which this vasculature can deliver drugs to the granuloma. Using anti-VEGF therapies to limit vascularization at the granuloma in rabbits, it was found that anti-VEGF despite reducing the degree of granuloma vascularization, enhanced delivery of small molecules to the granuloma by stabilizing the residual vasculature (132). In agreement with VEGF findings, the broad spectrum MMP inhibitor Marimastat was also found to stabilize the blood vessels of the granuloma in mice, leading to increased delivery of *Mtb* chemotherapeutics to the granuloma (135). Thus, variations in the degree vascularization and the organization and stabilization of granuloma vascularization are expected to influence accessibility of the lesion to mycobacterial chemotherapies.

After delivery to the granuloma *via* the vasculature, antimycobacterial therapies must cross the cellular layers that compose the granuloma and access *Mtb* within its niche in the granuloma. There are two distinct populations of bacteria within the granuloma, intracellular bacteria within macrophages and other immune cells and extracellular populations generally residing within the necrotic core of the granuloma. Antibiotic access to these disparate bacterial locations is driven by different considerations. In intracellular mycobacterial populations there is considerable variability in the degree to which individual antibiotics are able to enter and be retained by immune populations such as macrophages (136–139). Additionally, the metabolic changes driven in *Mtb* by the intracellular environment also alter the antibiotic susceptibility profile of *Mtb* (140–142). By contrast, for bacteria located within the central necrotic regions, antibiotics must not only penetrate the cellular layers of the granuloma but must also be able to diffuse efficiently into the caseum within the center of the granuloma (6, 143). Caseum in particular comprises a substantial barrier to antibiotic therapies, as many antibiotics either diffuse poorly into caseum or irreversibly bind to caseous material limiting deep penetration of antibiotics into the granuloma (136, 143, 144). The necrotic core of the granuloma and the composition of caseum itself also drives broad metabolic reprogramming of *Mtb* that make it resistant to many antibiotics (143, 145). Finally the reduced oxygen availability within the granuloma can also drive mycobacterial populations into a metabolically shifted, non-replicating state that can lead to altered susceptibility to antibiotics (146, 147).

The use of animal models with and without necrosis (148–150) as well as identification of spatial distribution of antibiotics with imaging mass spectrometry (136, 150–152) has shed light on the degree to which the complex structure of the tuberculous granuloma controls antibiotic access to *Mtb*. Consistent with their strong efficacy in the clinic, the frontline chemotherapeutics isoniazid and pyrazinamide effectively penetrate the outer cellular layers of the granuloma as well as the necrotic core in human patients and granuloma forming C3HeB/FeJ mice (136, 150). The other frontline therapies, rifampicin and ethambutol, were initially excluded from necrotic regions but concentrated within the necrotic core at later time points in either human lesions (rifampicin) or rabbit lesions (ethambutol) (136, 152). Despite the effective penetration of these antibiotics into the granuloma, experiments in granuloma-forming mouse models found that organized granuloma formation still limited the effectiveness of frontline antibiotics (148, 150), suggesting that the granuloma complicates treatment even for antibiotics with favorable distributions. Consistent with this, modeling of pharmacokinetic and pharmacodynamic data from humans found that many frontline and second-line therapies failed to reach desired concentrations in a subset of lesion types, with cavitary and caseous lesions being most difficult to treat (153).

Many second-line therapies also have limited diffusion into the core of necrotic granulomas (136, 137, 139, 150). Imaging of moxifloxacin in human and rabbit granulomas demonstrated that moxifloxacin penetrated the necrotic core far less efficiently than the cellular layers surrounding the core (136, 139). This asymmetric distribution of moxifloxacin and other fluoroquinolones was driven in part through strong uptake of this antibiotic by macrophage populations within the granuloma (139). While the clinical efficacy of moxifloxacin and pharmacodynamic studies demonstrate that moxifloxacin reaches effective concentrations within the granuloma (154), experiments in Nos2-deficient mice that form necrotic granulomas have suggested that the granuloma still serves as a barrier to effective therapy (148). Moxifloxacin treatment of Nos2-deficient animals prior to necrotic granuloma formation was approximately a log more effective in reducing bacterial burden than in animals that have already formed necrotic granulomas, consistent with granuloma necrosis limiting moxifloxacin efficacy (148). Similarly, while clinically equivalent moxifloxacin doses in rabbits were able to effectively clear bacterial burden in all types of granulomas, the use of suboptimal doses of moxifloxacin that mimic the case of patients with poor pharmacokinetics led to diminished clearance of bacteria within necrotic granulomas (154). By contrast, bacterial killing within cellular granulomas in these rabbits was equivalent for clinical and suboptimal dosing (154).

The complex structure of the granuloma also limits the use of other second-line therapies. Clofazimine is widely used to treat leprosy in clinical settings and has also been used against drug resistant *Mtb* (155, 156). However, Imaging of clofazimine localization in human patient samples revealed that although the drug penetrated the cellular layers of the granuloma very effectively, clofazimine was almost entirely excluded from the necrotic regions of the granuloma (136). Experiments in mouse models using the necrotic granuloma forming C3HeB/FeJ mice as well as BALB/c mouse models, which fail to form necrotic granulomas, found that while clofazimine was highly active in BALB/c mice, it was almost completely inactive in the necrotic granulomas that form in the lungs of C3HeB/FeJ mice (149). Further confirming that these differences in clofazimine effectiveness were likely related to granuloma structure, *Mtb* was found to be highly responsive to clofazimine in the spleens of C3HeB/FeJ mice, which lack necrotic granuloma formation (149). Taken together, these studies demonstrate that in *Mtb* infected individuals, the disparate features of the granuloma are a substantial barrier to therapy.

## Potential of Targeting Granuloma Formation as an Adjunctive Therapy

The central role of the granuloma in shaping host immune responses suggests that targeting the formation of granulomas could be used as a potential adjunctive therapy to facilitate productive immune responses within *Mtb* infected individuals. The disruption of the granuloma may serve to improve the ability of crucial cell types to recognize and eliminate mycobacteria within the granuloma either directly or through helper responses. Loss of granuloma integrity may enhance diffusion and recognition of mycobacterial products by the host immune system, potentially facilitating a more productive response within the host immune system. Similarly, loss of granuloma integrity may heighten the ability of host cytokines or immune molecules such as antibodies to access the bacteria and cells at the center of the granuloma. Beyond its effects on the host immune response, the granuloma also serves as a substantial barrier to the therapeutic efficacy of antibiotic treatments. Disruption of granulomas may increase the effectiveness of existing antimycobacterial therapies. Granuloma disruption could also enhance the spectrum of therapies that are effective against *Mtb*. Many chemotherapeutics are known to be effective against *Mtb in vitro* but have failed to translate into new clinical antibiotics. While the failure modes of these antibiotics are in many cases not fully understood, given the strong partitioning of some clinically effective anti-*Mtb* therapies, it is possible that at least some of these therapies that are effective *in vitro* fail due to limited penetration of these therapies in the context of organized granulomas. Thus, adjunctive therapeutic approaches targeting the granuloma may also facilitate the development of new antibiotics that are effective within disrupted granulomas.

While there is considerable promise in the development of adjunctive therapies targeting granuloma organization, there are also potential concerns of how granuloma disruption could alter disease outcomes during *Mtb* infection. In particular, the act of dissociating the granuloma may lead to the development of disseminated disease, a manifestation associated with poor disease outcome. Support for this idea comes from findings in HIV-positive individuals, where the degree of CD4 depletion is associated with disseminated disease and disruption of granuloma structure (157, 158). However, in contrast to findings in HIV-infected individuals, the granuloma has also been associated with dissemination in early infection (82, 83). A second potential limitation in targeting granuloma formation is that disruption of the granuloma and liberation of a large number of bacteria from the central core of the granuloma may lead to the host mounting an exuberant, host-detrimental response again *Mtb* in granuloma disrupted individuals. Excessive and problematic inflammatory responses are seen in *Mtb* infected, HIV-positive individuals, after beginning anti-retroviral therapy in a syndrome termed TB-immune reconstitution syndrome (TB-IRIS) (159). Finally, disruption of granulomas may enhance bacterial growth or allow the access to distinct niches within the infected host, potentially outweighing any advantages gained in antibiotic penetration and altered immune recognition.

While it remains to be seen whether there will be detrimental effects to therapies targeting granuloma structure, it may be possible to manage any potential drawbacks by timing the dissociation of granulomas. For instance, reduction of bacterial numbers through an initial period of antibiotic treatment may ultimately enable the safe disassembly of granulomas to improve subsequent therapies. Regardless, the ongoing difficulties in treating tuberculosis suggest that novel approaches to treating tuberculosis are worth pursuing. Adjunctively targeting the granuloma may enhance the effectiveness of existing treatments and shorten the duration of therapy required for these complex therapies. Granuloma dissociation could also enhance antimycobacterial immune responses, potentially boosting existing therapeutic approaches for both drug-sensitive and drug-resistant tuberculosis which could be further combined with immunomodulatory and antibiotic therapies. Lastly, targeting granuloma integrity may allow for the development of a wider palette of chemotherapeutics including drugs that fail to penetrate complex millieu of the organized granuloma. With the continued burden of tuberculosis worldwide and the increasing threat of drug-resistant *Mtb*, targeting of granuloma structure is a distinct approach to enhance the treatment of *Mtb* and is complementary to ongoing efforts to improve existing therapies and the development of new therapeutics. With the recent advances made in understanding granuloma formation and the molecular changes that underlie it, potential new points of intervention for therapies altering granuloma formation have been identified. Targeting these pathways may enable the rational development of new granuloma-targeted interventions.

## Author Contributions

The author confirms being the sole contributor of this work and has approved it for publication.

## Funding

This work was supported by the Max Planck Society.

## Conflict of Interest

The author declares that the research was conducted in the absence of any commercial or financial relationships that could be construed as a potential conflict of interest.

## Publisher’s Note

All claims expressed in this article are solely those of the authors and do not necessarily represent those of their affiliated organizations, or those of the publisher, the editors and the reviewers. Any product that may be evaluated in this article, or claim that may be made by its manufacturer, is not guaranteed or endorsed by the publisher.
